# Is Cesarean Delivery Preferable in Twin Pregnancies at >=36 Weeks Gestation?

**DOI:** 10.1371/journal.pone.0155692

**Published:** 2016-05-26

**Authors:** Yu Dong, Zhong-Cheng Luo, Zu-Jing Yang, Lu Chen, Yu-Na Guo, Ware Branch, Jun Zhang, Hong Huang

**Affiliations:** 1 Ministry of Education-Shanghai Key Laboratory of Children's Environmental Health, Xinhua Hospital, Shanghai Jiao-Tong University School of Medicine, Shanghai, China 200092; 2 Department of Obstetrics and Gynecology, Sainte-Justine Hospital, University of Montreal, Montreal, Quebec, Canada; 3 Department of Obstetrics and Gynecology, Xinhua Hospital, Shanghai Jiao-Tong University School of Medicine, Shanghai, China 200092; 4 Department of Obstetrics and Gynecology, International Peace Maternity and Child Health Hospital, Shanghai Jiao-Tong University School of Medicine, Shanghai, China 200030; 5 Department of Obstetrics and Gynecology, University of Utah and Intermountain Health Care, Utah, United States of America; Hospital de Especialidades del Niño y la Mujer de Queretaro, MEXICO

## Abstract

**Background:**

The optimal mode of delivery in twin pregnancies remains controversial. A recent randomized trial did not find any benefit of planned cesarean vs. vaginal delivery at 32–38 weeks gestation, but the trial was not powered to detect a moderate effect. We aimed to evaluate the impact of cesarean delivery on perinatal mortality and severe neonatal morbidity in twin pregnancies at ≥32 weeks through a large database exploration approach with the power to detect moderate risk differences.

**Methods:**

In a retrospective birth cohort study using the U.S. matched multiple births, 1995–2000 (the available largest multiple birth dataset), we compared perinatal outcomes in twins (n = 181,810 pregnancies) delivered at 32–41 weeks gestation without congenital anomalies. The primary outcome was a composite of perinatal death and severe neonatal morbidity. Cox regression was used to estimate the adjusted hazard ratio (aHR) controlling for the propensity to cesarean delivery, fetal characteristics (sex, birth weight, birth weight discordance, same-sex twin or not) and twin-cluster level dependence. Prospective risks were calculated using the fetuses-at-risk denominators.

**Results:**

The overall rates of the primary outcome were slightly lower in intended cesarean (6.20%) vs. vaginal (6.45%) deliveries. The aHRs of the primary outcome were in favor of vaginal delivery at 32 (aHR = 1.06, p = 0.03) or 33 (aHR = 1.22, p<0.001) weeks, neutral at 34–35 weeks, but in favor of cesarean delivery at 36 (aHR = 0.94, p = 0.004), 37, 38 and 39+ weeks (aHR: 0.72 to 0.78, all p<0.001). The lower risk of the primary outcome for cesarean vs. vaginal deliveries at 36+ weeks of gestation remained when the analyses were restricted to different-sex (dichorionic) twins (aHR = 0.84, 95% CI 0.80–0.88).

**Conclusion:**

Cesarean delivery may be beneficial for perinatal outcomes overall in twin pregnancies at ≥36 weeks gestation.

## Introduction

The prevalence of multifetal pregnancy, especially twin pregnancy, has increased over recent decades [1,2]. Compared to singleton pregnancies, twin pregnancies are at higher risk of maternal and neonatal complications such as gestational hypertension and preeclampsia, preterm birth, low birth weight, perinatal death, low 5-min Apgar score, neonatal seizures, and respiratory morbidity [3–7]. The optimal timing and method of delivery in twin pregnancies close to term remain controversial. A recent randomized trial in twin pregnancies showed that planned cesarean delivery did not significantly decrease or increase the risk of perinatal death or serious neonatal morbidity as compared to planned vaginal delivery at 32–38 weeks gestation in twin pregnancies with vertex presentation in the first twin [8]. However, the trial was powered to detect a large effect size only (a 50% reduction in a composite indicator of perinatal mortality and morbidity from 4.0% to 2.0%) [8]. The aim of the present study was to evaluate the impact of cesarean delivery on the risk of perinatal death and severe neonatal morbidity in twin pregnancies at 32–41 weeks gestation with vertex presentation in the first twin through a large database exploration approach with the power to detect moderate risk changes, taking into account the propensity to cesarean delivery. We hypothesized that the optimal method of delivery in twin pregnancies may be dependent on gestational age.

## Methods

### Study design

This was a retrospective cohort study of twin births, using the U.S. National Center for Heath Statistics (NCHS)’s 1995–2000 matched multiple birth dataset (the largest available linked multiple birth dataset) [9]. The NCHS used the matching certificate numbers to extract records from the NCHS statistical data files. The methodological details of the data linkage of birth and death records are available in the Technical Notes of National Vital Statistical Reports [10].

Available information in the linked multiple birth database included maternal demographic characteristics (e.g. race, age), obstetric history, current pregnancy complications, fetal presentation, labor problems (e.g. dysfunctional labor), mode of delivery, birth order, birth outcomes (e.g., sex, gestational age, birth weight), severe newborn complications (e.g., 5-min Apgar score <4), and infant survival status up to 1 year of age. Reported gestational age in completed weeks (used in the NCHS publications) was primarily based on the date of last menstruation; clinical estimates of gestational age (where available) were used if gestational age differed by > = 2 weeks in the two methods. The NCHS birth database contained 21 items for reporting 20 specific and “other” congenital anomalies. These fields were used to capture and exclude twin pairs with any reported congenital anomaly in either twin (first- or second-born).

**Fig 1:**
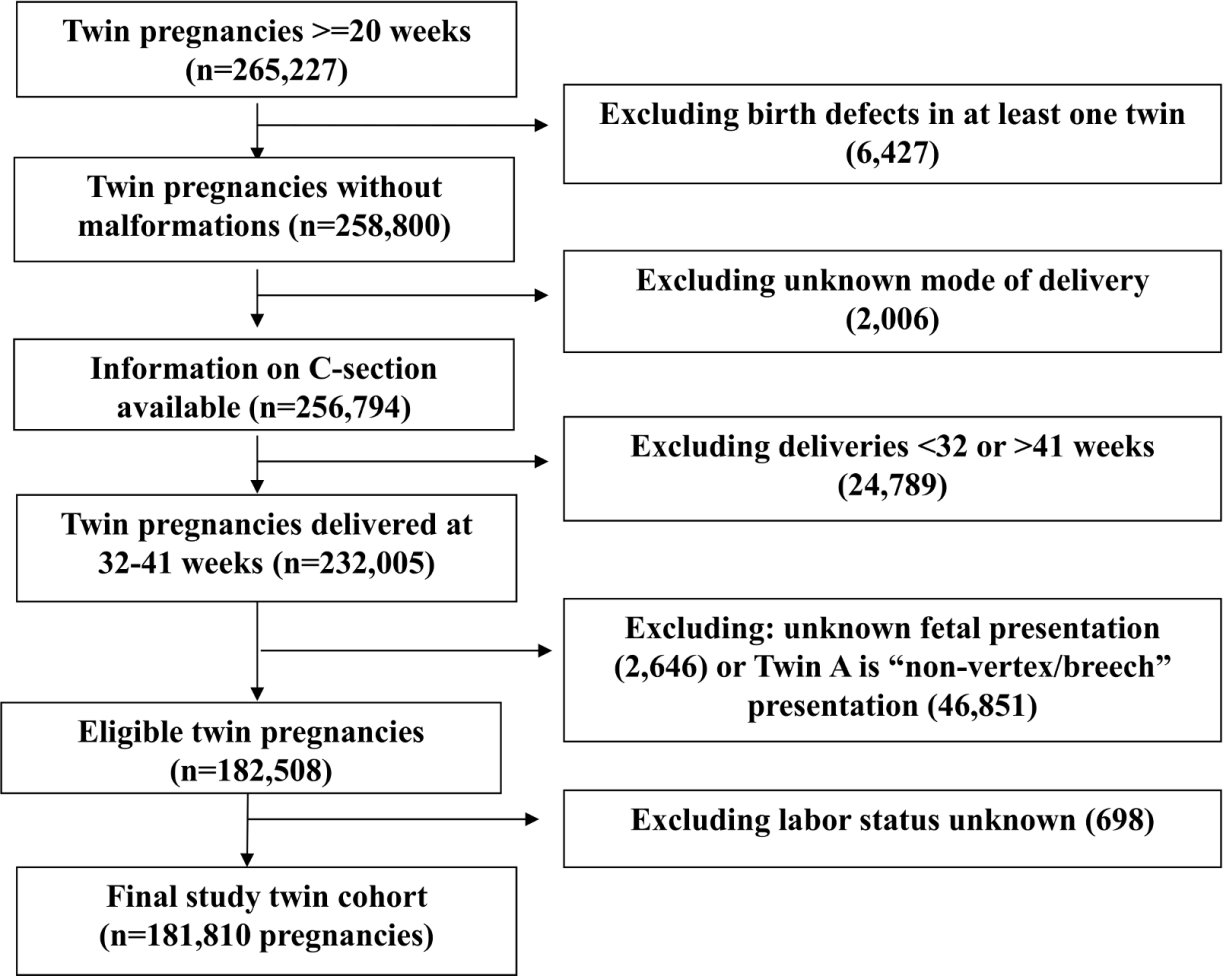
Flow chart in the selection of the twin pregnancy study cohort, the U.S. matched multiple birth data, 1995–2000.

The selection of the twin study cohort (n = 181,810 twin sets) is presented in **Fig 1.** We studied twin births at 32–41 weeks gestation because planned delivery before 32 weeks is not recommended unless there is a very strong clinical risk indication to fetuses, and deliveries at >41 weeks are rare for twins. Of a total of 265,227 twin pregnancies, we excluded twin sets with: 1) birth defects in at least one twin (n = 6,427); 2) unknown mode of delivery (n = 2,006); 3) deliveries at <32 weeks or >41weeks gestation (n = 24,789); 4) unknown fetal presentation (n = 2,646) or “non-vertex/breech” presentation in the first twin (n = 46,851) (because non-vertex/breech presentation in the first-presenting twin necessitates cesarean delivery in most obstetric practices); and 5) unknown labor status (n = 698). The presentation of the first baby of the twin is an important consideration in choosing the mode of delivery since vaginal delivery is not recommended if the first twin is in “non-vertex/breech” presentation. Sensitivity analyses were conducted to check whether the results were similar if the analyses were restricted to twin sets with vertex presentation in both twins. Because it is impossible to distinguish between intrapartum vs antepartum fetal deaths in the database, sensitivity analyses were conducted to examine whether the findings were similar if stillbirths were excluded from the analyses.

### Ethics statement

Ethical approval was exempted by the Research Ethics Board of Xinhua Hospital, Shanghai Jiao-Tong University, since the study was based on the anonymized NCHS matched multiple birth database publically available (http://www.cdc.gov/nchs/nvss/birth_methods.htm). The authors had no access to identifying information in study subjects.

### Intended mode of delivery

**Fig 2:**
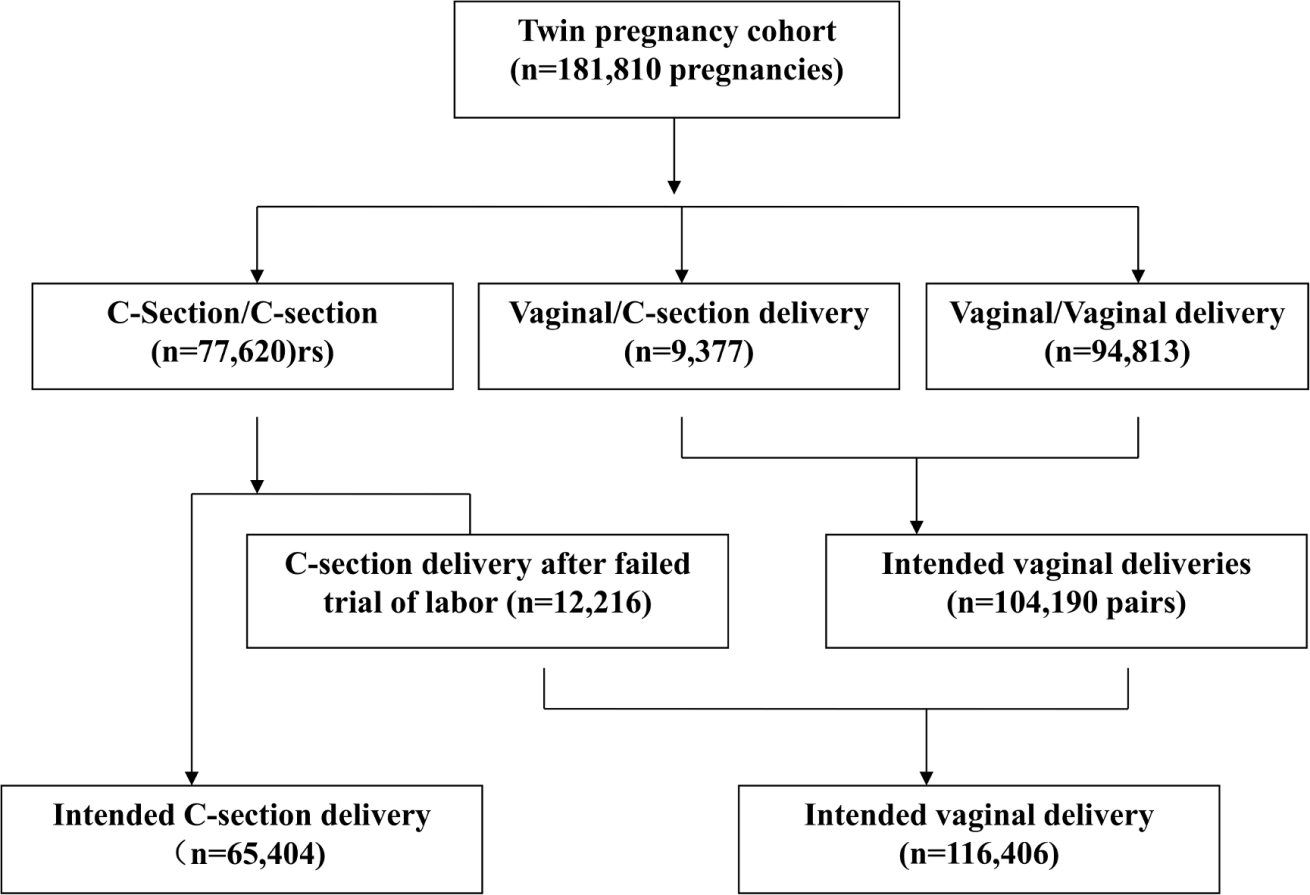
Classifications of intended mode of delivery, based recorded mode of delivery in twin pairs (Twin A-Twin B) and evidence of trial of labor in the twin pregnancy study cohort.

We classified twin sets by “intended” mode of delivery based on the reported mode of delivery in twin pairs and the presence of labor problems (**Fig 2**). Twin sets with cesarean delivery for both twins were classified as intended cesarean delivery. The NCHS multiple birth dataset contained variables on induction or stimulation of labor (missing 0.8%), prolonged (defined as >20 hours), or dysfunction labor (yes/no) (missing 1.0%). Cesarean deliveries with any recorded labor problems, including prolonged or dysfunctional labor or induction or stimulation of labor (thus indicating a trial of labor before cesarean section, n = 12,216), were classified as “intended vaginal delivery”. There were 9,377 twin pairs of first twin vaginal-second twin cesarean births. Because it is unlikely that second twin’s cesarean delivery following first twin’s vaginal delivery is a planned event, all such twin sets were classified as intended vaginal deliveries. The final study twin cohort included 65,404 intended cesarean deliveries (36.0%) and 116,406 cases intended vaginal deliveries (64.0%).

### Outcomes

The primary outcome was a composite of perinatal mortality or serious neonatal morbidity. Perinatal mortality included fetal deaths ≥20 weeks gestation and neonatal deaths in the first 28 days postpartum. Serious neonatal morbidity was defined as one or more of the followings: low 5-min Apgar score (less than 4), birth injury/trauma, meconium aspiration syndrome, need for assisted ventilation, and neonatal seizures (within 72 hours of age) as recorded in the linked birth data. The secondary outcomes were individual components of the primary outcome.

### Statistical analysis

Propensity score was calculated by a logistic regression model predicting the probability of cesarean delivery given the values of measured risk factors for cesarean delivery (maternal characteristics and pregnancy complications). Prospective risks (rates) of adverse outcomes were calculated using the fetuses-at-risk denominators (including unborn fetuses). Cox regression models weighted by the inverse of the probability of treatment (cesarean delivery) were used to account for the propensity to cesarean delivery in calculating the adjusted hazard ratio (aHR) and 95% confidence intervals of the composite primary outcome and its individual components comparing intended cesarean vs. vaginal delivery. The time variable was gestational age. The aHRs were controlled for the propensity to cesarean delivery (through weighting by the inverse of the propensity score), important known fetal characteristics [sex, birth weight, birth weight discordance or not (discordance according to the commonly used definition: birth weight difference >25% in a twin pair), and same-sex twin pair or not] and twin-cluster level dependence. The propensity score of cesarean delivery was estimated by logistic regression using all available risk factors of cesarean delivery. We evaluated the changes in the risk (aHR) of the composite primary outcome comparing cesarean vs vaginal deliveries over gestational age in twins overall, and for first and second twins (according to delivery order) separately. All data analyses were conducted using SAS 9.2 (North Carolina, USA). P values <0.005 were considered statistically significant, considering 9 primary comparisons of interest (comparisons of the primary outcome overall, and at each gestational week from 32 to 39) (0.05/9 = 0.0056). The study sample size (n = 181,810 pregnancies) had a power of >90% to detect a 20% reduction in the risk of the composite primary outcome (perinatal death or serious newborn morbidity) overall or at specific gestational week from 32 to 39 weeks.

## Results

There were 65,404 twin pregnancies (130,808 fetuses/infants) in the cesarean delivery group, and 116,406 twin pregnancies (232,812 fetuses/infants) in the vaginal delivery group. Maternal characteristics were significantly different in cesarean vs. vaginal delivery groups, although most of those differences were small (**Table 1)**. Women in the cesarean delivery group were more likely to be ≥35 y of age or primiparous, but less likely to be a smoker. Though statistically significant, these differences in cesarean delivery rates by maternal characteristics were mostly small.

**Table 1:** Maternal characteristics by intended mode of delivery * in twin pregnancies without malformations (n = 181,810), U.S. 1995–2000.

	C-Section	Vaginal	C-section	Crude	
	N (%)	N (%)	rate (%)	RR (95CI)	P
N	65,404	116,406			
Age, years					<0.001
<20	3,647 (5.6)	7,824 (6.7)	31.8	0.86(0.83–0.90)	
≥35	14,343 (21.9)	20,804 (17.9)	40.8	1.28(1.25–1.31)	
20–34	47,414 (72.5)	87,778 (75.4)	35.1	Reference	
Race					0.005
White	51,867 (79.3)	93,057 (79.9)	35.8	0.96(0.91–1.01)	
Black	10,833 (16.6)	18,703 (16.1)	36.7	1.00(0.94–1.05)	
Others	2,704 (4.1)	4,646 (4.0)	36.8	Reference	
Education (missing: n = 666)					<0.001
<High school	9,914 (15.2)	17,250 (14.8)	36.5	1.06(1.03–1.09)	
High school	19,470 (29.8)	34,560 (29.7)	36.0	1.04(1.02–1.07)	
College	15,378 (23.5)	26,399 (22.7)	36.8	1.07(1.05–1.10)	
University	19,976 (30.5)	36,871 (31.7)	35.1	Reference.	
Parity					<0.001
Primiparous	47,995 (74.4)	84,031 (72.2)	36.4	1.06(1.04–1.09)	
Multiparous	17,409 (26.6)	32,375 (27.8)	35.0	Reference	
Smoking					<0.001
Unknown/missing	14,188 (21.7)	19,438 (16.7)	42.2	1.38(1.34–1.41)	
Smoking	4,959 (7.6)	9,674 (8.3)	33.9	0.97(0.93–1.00)	
Non-smoking	46,257 (70.7)	87,294 (75.0)	34.6	Reference	

Data presented are n (% in column) of pregnancies.

C-section = cesarean section; RR = relative risk; CI = confidence interval.

**Table 2**presents the rates of cesarean delivery by medical risk factors and obstetric complications. Diabetes, chronic or pregnancy-induced hypertension, eclampsia, cephalopelvic disproportion, or fetal distress was associated with a small-to-moderately increased risk of cesarean delivery (RR: 1.15 to 1.58), while abruptio placenta or placenta previa was associated with a substantially increased risk of cesarean delivery (RR: 2.77 to 7.34).

**Table 2:** Medical risk factors, obstetric complications and cesarean-section delivery rates in twin pregnancies.

	C-section*	Vaginal delivery*	Crude	
	N = 65,404	N = 116,406	RR (95CI)	P
Anemia (missing n = 804)				
yes	1,701 (32.59)	3,518 (67.41)	0.86 (0.81–0.91)	<0.001
no	63,424 (36.08)	112,363 (63.92)	Reference	
Diabetes (missing n = 804)				
yes	2,377 (39.96)	3,572 (60.04)	1.19 (1.13–1.26)	<0.001
no	62,748 (36.65)	112,309 (64.16)	Reference	
Chronic	hypertension	(missing n = 804)		
yes	681 (42.14)	935 (57.86)	1.30 (1.18–1.44)	<0.001
no	64,444 (35.92)	114,946 (64.08)	Reference	
Pregnancy-associated hypertension (missing n = 804)				
yes	5,800 (39.02)	9,063 (60.98)	1.15 (1.11–1.19)	<0.001
no	59,325 (35.71)	106,818 (64.29)	Reference	
Eclampsia	(missing n = 804)			
yes	667 (42.46)	904 (57.54)	1.32 (1.19–1.46)	
no	64,458 (35.92)	114,977 (64.08)	Reference	
Premature rupture of membrane				
yes	2,444 (29.43)	5861 (70.57)	0.73(0.70–0.77)	<0.001
no	62,960 (36.29)	110,545 (63.71)	Reference	
Abruptio placenta				
yes	814 (60.66)	528 (39.34)	2.77(2.48–3.09)	<0.001
no	64,590 (35.79)	115,878 (64.21)	Reference	
Placenta previa				
yes	606 (80.37)	148 (19.63)	7.34(6.13–8.79)	<0.001
no	64,798 (35.79)	116,258(64.21)	Reference	
Cephalopelvic	Disproportion (missing n = 13)			
yes	747 (42.61)	1,006 (57.39)	1.33(1.21–1.46)	<0.001
no	64,653 (35.91)	115,391 (64.09)	Reference	
Cord prolapsed				
yes	253 (44.62)	314 (55.38)	1.44(1.22–1.70)	<0.001
no	65,151 (35.95)	116,092 (64.05)	Reference	
Fetal distress (missing n = 10,411)				
yes	2,466 (46.18)	2,874 (53.82)	1.58(1.50–1.67)	<0.001
no	58,408 (35.17)	107,651 (64.83)	Reference	

*Data presented are n (% in row) of pregnancies.

The propensity score to cesarean section for each delivery was calculated in a logistic regression model that included all the risk factor variables of cesarean delivery presented in Tables 1 and 2. The mean, median, minimal, maximal, 25^th^ and 75^th^ percentile values of the propensity score were 0.3552, 0.3348, 0.1697, 0.9522, 0.3077 and 0.3900 in the study cohort, respectively.

**Table 3**presents the risks of the composite primary outcome (perinatal death or any severe neonatal morbidity) overall and its individual components comparing cesarean vs vaginal delivery. The crude rates showed a slightly lower rate of the primary outcome in cesarean deliveries (6.20% vs. 6.45%), but the adjusted HR showed a stronger protective effect (aHR = 0.93, p<0.001). Cesarean delivery was associated with a lower risk of birth injuries (aHR = 0.26, p<0.001), low 5-min Apgar score (adjusted HR = 0.84, p<0.001), and assisted ventilation (aHR = 0.94, p<0.001), but a slightly higher risk of perinatal death (aHR = 1.12, p = 0.001) overall mostly attributable to higher risk of stillbirth (aHR = 1.21, p<0.001). There were no significant differences in the risks of meconium aspiration syndrome or neonatal seizures.

**Table 3:** Adverse perinatal outcomes comparing cesarean-section (n = 130,808 infants) vs. vaginal (n = 232,812 infants) deliveries in twin pregnancies.

		Crude		Adjusted ^b^	
C-section*	Vaginal*	HR (95CI)	P	HR (95CI)	P
**Composite adverse perinatal outcome** ^**a**^					
8,115(6.20)	15,013(6.45)	1.03(1.00–1.06)	0.05	0.93(0.92–0.95)	<0.001
**Composite neonatal severe morbidity** ^**a**^ (missing n = 844)					
7,627(5.85)	14,316(6.16)	1.01(0.99–1.04)	0.38	0.92(0.90–0.94)	<0.001
5-min Apgar score <4 (missing n = 69,906)					
242(0.24)	526(0.27)	0.98(0.84–1.14)	0.76	0.84(0.76–0.92)	<0.001
Birth injury (missing n = 33,171)					
72(0.06)	508(0.24)	0.28(0.22–0.36)	<0.001	0.26(0.22–0.30)	<0.001
Meconium aspiration syndrome (missing n = 6,961)					
87(0.07)	153(0.07)	1.10(0.84–1.43)	0.48	1.02(0.87–1.21)	0.78
Neonatal seizures (missing n = 6,961)					
66(0.05)	145(0.06)	0.87(0.65–1.17)	0.37	0.84(0.70–1.00)	0.05
Assisted ventilation (missing n = 19,156)					
7,323(5.90)	13,403(6.08)	1.03(1.01–1.06)	0.02	0.94(0.93–0.96)	<0.001
**Perinatal death**					
573(0.44)	816(0.35)	1.33(1.20–1.48)	<0.001	1.12(1.04–1.19)	0.001
Stillbirth					
339(0.26)	505(0.22)	1.27(1.11–1.46)	<0.001	1.21(1.10–1.33)	<0.001
Neonatal	death				
234(0.18)	311(0.13)	1.43(1.20–1.69)	<0.001	1.14(1.02–1.28)	0.02

*Data presented are n (%) for each outcome in infants of cesarean and vaginal deliveries; the unit of analysis is the infant.

^a^ Composite adverse perinatal outcome: perinatal death or any severe neonatal morbidity listed in the Table; composite neonatal morbidity: any severe neonatal morbidity listed in the Table.

^b^ Hazard ratios from Cox regression models adjusting for the propensity to C-section (through weighting by the inverse of the propensity score), birth weight, birth weight discordance (>25%) in twins, infant sex, same-sex twin or not, and twin cluster-level dependence; the unit of analysis is the infant.

Gestational-age specific prospective risks of adverse perinatal outcomes comparing intended cesarean vs. vaginal delivery in twins are presented in **Table 4.** Comparing cesarean vs. vaginal deliveries adjusting for the propensity score to cesarean section and fetal characteristics, there were slightly higher risks of the composite adverse perinatal outcomes for cesarean twin deliveries at 32–33 weeks (adjusted HRs: 1.06 to 1.18), similar risks at 34–35 weeks (p>0.05), but lower risks at 36, 37, 38 or 39+ weeks gestation (adjusted HRs: 0.72 to 0.94, all p<0.005). A similar pattern was observed for the occurrence of anyone or more of the reported severe neonatal morbidities. The aHRs for perinatal death comparing cesarean vs. vaginal delivery fluctuated, showing higher risks at 33, 36, and 38 weeks gestation, a slightly lower risk at 37 weeks, but similar risks at 32, 35, or 39+ weeks gestation. These risk differences at most gestational weeks were not statistically significant after accounting for multiple tests. For births at ≥36 weeks overall, there was a 20% lower risk of the composite adverse perinatal outcome (adjusted HR = 0.80, p<0.001), and a 22% lower risk of the composite neonatal severe morbidity (adjusted HR = 0.78, <0.001).

**Table 4:** Gestational-age specific risks* of adverse perinatal outcomes comparing cesarean-section (130,808 infants) vs. vaginal (n = 232,812 infants) deliveries in twin pregnancies.

Gest. age	C-section	Vaginal	Crude		Adjusted ^b^	
(weeks)	delivery*	delivery*	HR (95CI)	P	HR (95CI)	P
**Composite adverse perinatal outcome** ^a^**, n (%)**						
32	994 (0.76)	1,284 (0.55)	1.38(1.27–1.50)	<0.001	1.06(1.01–1.11)	0.028
33	1,207 (0.97)	1,560 (0.69)	1.40(1.30–1.51)	<0.001	1.18(1.13–1.24)	<0.001
34	1,264 (1.09)	1,993 (0.94)	1.17(1.09–1.25)	<0.001	1.04(1.00–1.09)	0.075
35	1,192 (1.16)	2,201 (1.15)	1.02(0.95–1.09)	0.67	0.96(0.92–1.01)	0.098
36	1,200 (1.42)	2,380 (1.47)	0.97(0.90–1.04)	0.34	0.94(0.90–0.98)	0.004
37	951 (1.53)	2,167 (1.80)	0.85(0.79–0.92)	<0.001	0.78(0.74–0.81)	<0.001
38	670 (1.77)	1,669 (2.22)	0.80(0.73–0.87)	<0.001	0.72(0.68–0.76)	<0.001
39+	637 (3.58)	1,758 (4.58)	0.76(0.70–0.84)	<0.001	0.72(0.68–0.76)	<0.001
≥36	3,458(4.08)	7,974(4.91)	0.86(0.82–0.89)	<0.001	0.80(0.78–0.82)	<0.001
**Composite neonatal severe morbidity,** ^a^ **n (%)**						
32	926 (0.71)	1,202 (0.52)	1.37(1.26–1.49)	<0.001	1.06(1.00–1.11)	0.05
33	1,130 (0.91)	1,459 (0.65)	1.40(1.30–1.51)	<0.001	1.19(1.14–1.25)	<0.001
34	1,193 (1.03)	1,909 (0.90)	1.15(1.07–1.24)	0.0002	1.03(0.98–1.07)	0.23
35	1,122 (1.10)	2,083 (1.08)	1.01(0.94–1.09)	0.77	0.96(0.91–1.00)	0.05
36	1,133 (1.34)	2,301 (1.42)	0.95(0.88–1.02)	0.12	0.91(0.87–0.96)	<0.001
37	909 (1.46)	2,073 (1.72)	0.85(0.78–0.92)	<0.001	0.77(0.74–0.81)	<0.001
38	629 (1.66)	1,612 (2.14)	0.78(0.71–0.85)	<0.001	0.69(0.65–0.74)	<0.001
39+	585 (3.29)	1,677 (4.37)	0.73(0.67–0.81)	<0.001	0.70(0.66–0.74)	<0.001
≥36	3,256(3.85)	7,663(4.73)	0.84(0.81–0.87)	<0.001	0.78(0.76–0.80)	<0.001
**Perinatal death, n (%)**						
32	83 (0.06)	103 (0.04)	1.43(1.07–1.92)	0.01	0.99(0.83–1.19)	0.95
33	96 (0.08)	107 (0.05)	1.62(1.23–2.14)	<0.001	1.23(1.03–1.46)	0.02
34	80 (0.07)	98 (0.05)	1.50(1.12–2.02)	<0.001	1.27(1.06–1.54)	0.01
35	79 (0.08)	135 (0.07)	1.10(0.83–1.45)	0.51	1.04(0.88–1.23)	0.64
36	77 (0.09)	92 (0.06)	1.61(1.19–2.17)	0.002	1.52(1.26–1.83)	<0.001
37	51 (0.08)	109 (0.09)	0.91(0.65–1.26)	0.55	0.79(0.63–0.99)	0.038
38	45 (0.12)	65 (0.09)	1.38(0.94–2.01)	0.10	1.34(1.06–1.70)	0.014
39+	62 (0.35)	107 (0.28)	1.22(0.89–1.66)	0.22	0.92(0.76–1.12)	0.41
≥36	235(0.28)	373(0.23)	1.25(1.06–1.47)	0.008	1.11(1.00–1.23)	0.05

*Data presented are n (%); prospective risks (rates) were calculated using the fetuses-at-risk denominators; unborn fetuses were censored and included in the denominators (e.g. for calculating the outcome rates at 37 weeks, infants born after 37 weeks were censored and included in the denominators); the unit of analysis is the infant.

^a^ Composite adverse perinatal outcome: perinatal death or any severe neonatal morbidity listed in Table 3; composite neonatal severe morbidity: any severe neonatal morbidity listed in Table 3.

^b^ Hazard ratio from Cox regression models adjusting for the propensity to C-section (through weighting by the inverse of the propensity score), birth weight, birth weight discordance (>25%) in twins, infant sex, same-sex twin or not, and twin cluster-level dependence; the unit of analysis is the infant.

**Fig 3:**
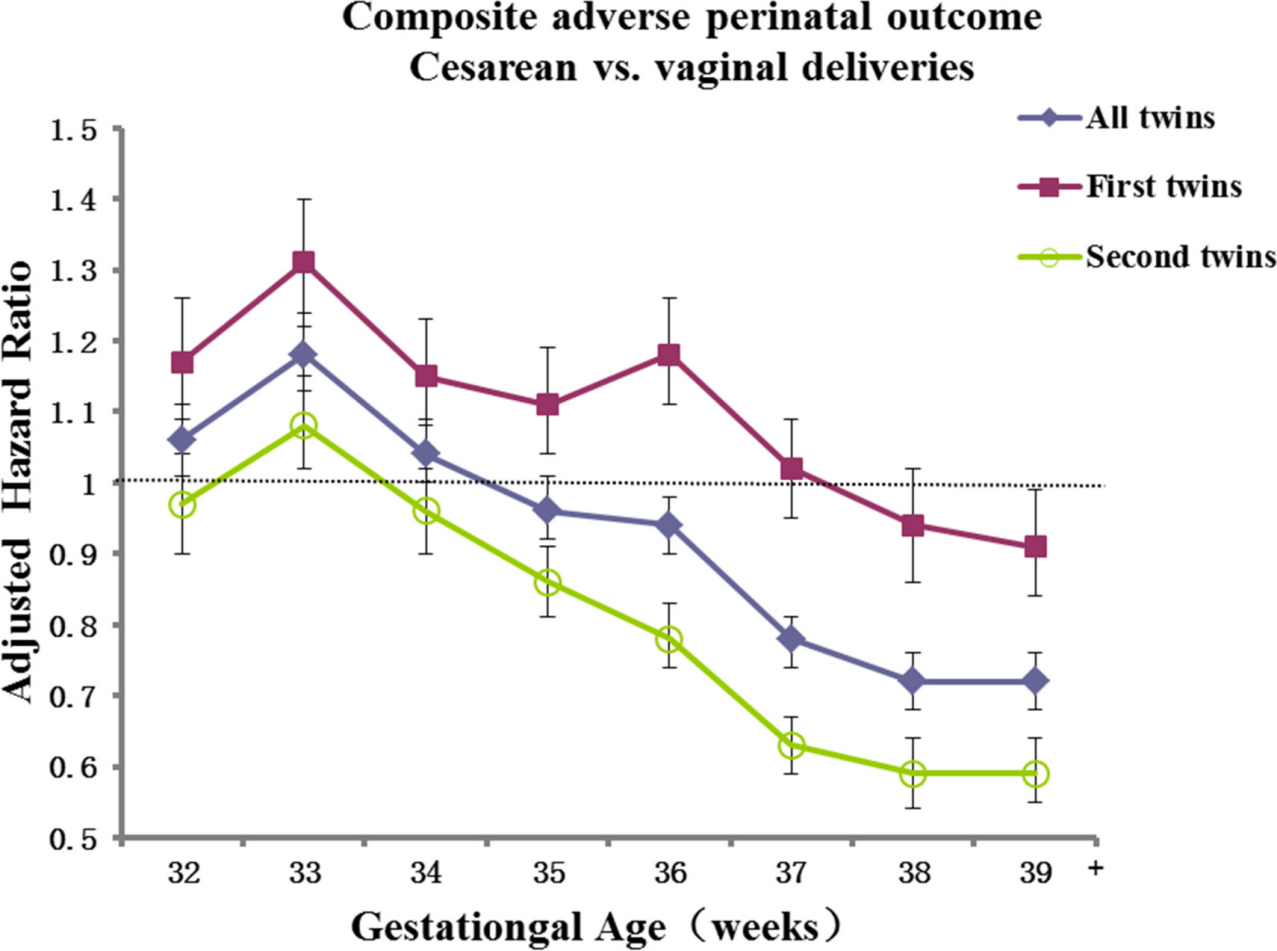
The adjusted hazard ratios of the composite primary outcome (perinatal death or severe neonatal morbidity) comparing cesarean vs vaginal deliveries over gestational age (weeks) in all twins, first twins and second twins. The hazard ratios were from Cox regression models adjusting for the propensity to C-section (through weighting by the inverse of the propensity score), birth weight, birth weight discordance (>25%) in twins, infant sex, same-sex twin or not, and twin cluster-level dependence; the unit of analysis is the infant. The error bars represent the 95% confidence intervals.

Gestational age-specific adjusted HRs showed that the overall beneficial effect of cesarean delivery against adverse perinatal outcomes in all twins at over 36 weeks of gestation was mainly due to the protective effect for second twins (**Fig 3**).

If the analyses were restricted to twin births with vertex presentation in both twins (n = 311,748 babies), similar findings were observed. The crude rates showed a slightly lower risk of the primary outcome in cesarean vs. vaginal delivery (5.82% vs. 5.91%, aHR = 0.96, p<0.001). Gestational age-specific adjusted HRs showed that the risk of the composite adverse perinatal outcome for cesarean delivery was slightly higher for twin births at 32–34 weeks (aHRs: 1.06 to 1.10), similar at 35–36 weeks (p>0.05), but lower at 37, 38 or 39+ weeks gestation (aHRs: 0.73 to 0.82, all p<0.001).

If stillbirths were excluded from the analyses, similar findings for the primary outcome were observed. Gestational age-specific adjusted HRs showed that the risk of the composite adverse outcome for cesarean delivery was higher for twin births at 32–34 weeks (aHRs: 1.07 to 1.23, all p<0.005), similar at 35–36 weeks (aHRs: 0.96 to 1.00, all p>0.05), but lower at 37, 38 or 39+ weeks gestation (aHRs: 0.73 to 0.81, all p<0.001).

Because “assisted ventilation” accounted for a large portion of the composite primary outcome, we conducted sensitivity analyses excluding assisted ventilation from the composite primary outcome. A similar but stronger overall protective effect of cesarean delivery was observed: a lower rate of the primary outcome was observed in cesarean vs. vaginal deliveries (0.74% vs. 0.86%, aHR = 0.81, p<0.001). Gestational age-specific aHRs showed that the risk of the primary outcome for cesarean delivery was lower for twin births at 35 (aHR = 0.80, p<0.001), 37, 38, or 39+ weeks gestation (aHRs: 0.59 to 0.68, all p<0.001), and non-significantly lower at 36 weeks (aHR = 0.91, p = 0.14). There were no significant risk differences at 32–34 weeks gestation (data not shown).

Because chorionicity is an important risk factor for adverse perinatal outcomes, and we did not have direct information on chorionicity in the database, we conducted sensitivity analyses by restricting the analyses to non-malformation different-sex (thus dichorionic) twin pregnancies with vertex presentation in both twins and without major maternal pathologies (diabetes, hypertension and eclampsia). Similar results were observed (**Table 5**). The risks of adverse perinatal outcomes were similar in cesarean vs. vaginal deliveries for twins delivered at 32–35 weeks gestation (aHR = 1.01, 95% CI 0.96–1.05), but lower in cesarean deliveries for twins delivered at 36+ weeks gestation (aHR = 0.84, 95% CI 0.80–0.88).

**Table 5:** Composite adverse perinatal outcome comparing cesarean-section (n = 41,020 infants) vs. vaginal (n = 71,804 infants) deliveries in the analyses restricted to different-sex (dichorionic) twin pregnancies without major maternal pathologies^a^.

Gest. age	C-section	Vaginal	Crude		Adjusted ^b^		Adjusted ^c^	
(weeks)	delivery	delivery	HR [95CI]	P	HR [95CI]	P	HR [95CI]	P
	n (%)	n (%)						
32	226(0.55)	391(0.54)	1.01(0.86–1.19)	0.89	0.84(0.71–0.99)	0.04	0.85(0.72–1.01)	0.06
33	295(0.75)	425(0.61)	1.23(1.06–1.43)	0.006	1.10(0.94–1.27)	0.24	1.02(0.94–1.12)	0.60
34	348(0.95)	558(0.85)	1.12(0.98–1.28)	0.11	1.03(0.90–1.17)	0.70	1.01(0.93–1.09)	0.86
35	376(1.14)	598(1.00)	1.14(1.01–1.30)	0.04	1.11(0.97–1.26)	0.13	1.11(1.03–1.20)	0.007
36	344(1.26)	687(1.35)	0.93(0.82–1.06)	0.30	0.92(0.81–1.05)	0.23	0.92(0.86–1.00)	0.04
37	285(1.43)	661(1.74)	0.82(0.71–0.94)	0.005	0.81(0.71–0.94)	0.004	0.81(0.75–0.88)	<0.001
38	206(1.71)	493(2.08)	0.82(0.70–0.97)	0.02	0.81(0.69–0.95)	0.01	0.80(0.72–0.88)	<0.001
39+	209(3.79)	511(4.25)	0.86(0.73–1.01)	0.07	0.82(0.70–0.97)	0.02	0.81(0.73–0.89)	<0.001
32–35	1,245(3.04)	1,972(2.75)	1.13(1.05–1.21)	<0.001	1.03(0.96–1.10)	0.45	1.01(0.96–1.05)	0.06
≥36	1,044(3.83)	2,352(4.62)	0.86(0.80–0.93)	<0.001	0.85(0.79–0.91)	<0.001	0.84(0.80–0.88)	<0.001
All	2,289(5.58)	4,324(6.02)	0.99(0.94–1.04)	0.63	0.94(0.89–0.99)	0.01	0.93(0.90–0.95)	<0.001

^a^ The comparisons on composite adverse perinatal outcome (perinatal death or any severe neonatal morbidity) were in different-sex non-malformation twin pregnancies without major maternal pathologies (diabetes, hypertension and eclampsia) with vertex presentation in both twins.

^b^ Hazard ratios comparing C-section vs. vaginal delivery adjusting for birth weight, birth weight discordance (>25%) in twins, sex of the baby, and twin cluster-level dependence; the unit of analysis is the infant.

^c^ Hazard ratios further adjusting for the propensity score to C-section, in additional to the variables adjusted in the above model.

We further assessed whether second twins were at higher risk of the composite primary outcome than first twins by mode of delivery. In vaginal deliveries, second twins were at higher risk of the composite adverse perinatal outcome than first twins (aHR = 1.33, 95% CI: 1.28–1.37). In contrast, in cesarean deliveries, second twins were at slightly lower risk of the composite adverse perinatal outcome than first twins (aHR = 0.94, 95% CI: 0.90–0.98).

## Discussion

### Main findings

In this large twin birth cohort study, we found that cesarean delivery was associated with better perinatal outcomes overall in twin pregnancies at ≥36 weeks, taking into account fetal characteristics and the propensity to cesarean delivery. This is mainly due to a lower risk of severe neonatal morbidities. The beneficial effect of cesarean delivery against adverse perinatal outcomes in twins overall is mainly attributable to the protective effect for second twins.

### Comparisons with previous studies

A recent randomized trial found no significant increase or decrease in the risk of a composite adverse perinatal outcome (perinatal death or any severe neonatal morbidity) comparing planned cesarean vs. vaginal delivery in twin pregnancies with vertex presentation in the first-twin at 32–38 weeks gestation [8]. The severe neonatal morbidities observed in the study were somewhat similar to those in our study and included birth trauma, 5-min Apgar score <4, abnormal level of consciousness, neonatal seizures, assisted ventilation, neonatal sepsis, necrotizing enterocolitis, and cystic periventricular leukomalacia. However, we used the linked administrative birth-related health data with a smaller number of severe neonatal morbidity conditions (no data on abnormal level of consciousness, neonatal sepsis, necrotizing enterocolitis, or cystic periventricular leukomalacia; some of these patients likely had low 5-min Apgar score and thus might have already been included). Analysis of our data showed that adverse perinatal outcomes occurred less frequently in cesarean deliveries at 36+ gestational weeks. There were likely under-reportings of severe neonatal morbidities in administrative health data. Such under-reportings were most likely non-differential to mode of delivery, and might not have affected the comparisons.

We calculated gestational age-specific prospective hazard ratios of adverse outcomes (considering all fetuses-at-risk as the denominators including unborn fetuses), while gestational age group-specific odds ratios (risks) were calculated in the recent trial (conditional risk on delivery, unborn fetuses were excluded in the denominators) showing no effect of planned method of delivery [8]. The prospective, fetuses-at-risk approach assumed that the intended mode of delivery was the same as the actual mode of delivery. However, if a pregnancy complication occurred in a woman with planned vaginal delivery, the woman might switch to planned cesarean delivery. Such “cross-overs” would only tend to shift more high-risk women to the cesarean group, and therefore would bias the results in favor of vaginal, rather than cesarean delivery. Furthermore, sensitivity analyses showed that if we applied logistic regression to model the gestational age-specific risks excluding unborn fetuses, the results would still be in favor of cesarean delivery.

Our study findings support most previous observational studies showing that planned cesarean delivery was associated with lower risk of serious neonatal morbidity than intended vaginal delivery close to term [11–13]. However, our study is much larger in sample size, thus allowing gestational week-specific risk estimates. Also, we have accounted for the propensity to cesarean section (not in previous observational studies).

A recent study of 193 twins with low birth weight found that vaginal delivery was associated with an increased risk of intraventricular hemorrhage [14]. Unfortunately, we did not have data on intraventricular hemorrhage. A French study of 758 twins suggested that vaginal delivery may be a safe option for twin pregnancies with a cephalic-presenting first twin at 35+ weeks gestation [15]. In contrast, analysis of our large twin cohort data suggest that cesarean delivery is preferable at 36+ weeks.

An observational study of 8073 twin births in Scotland found that planned cesarean delivery may substantially reduce the risk of perinatal death of twins at or after 36 weeks of gestation [16]. This finding could not be confirmed in our large twin cohort. We observed no significant difference in the risk of perinatal death comparing cesarean vs. vaginal deliveries at ≥36 weeks overall. In addition, there was a higher risk of perinatal death for cesarean deliveries at exactly 36 weeks even after accounting for multiple tests, probably due to commonly planned cesarean delivery at 36 weeks for uncaptured high-risk conditions. Also, our results confirmed the previous finding that second twins were at higher risk of adverse perinatal outcomes in vaginal deliveries [16].

### Strengths and limitations

The main strength of our study is the large sample size, allowing the power to detect gestational age-specific, moderate risk changes. Our study has limitations. Firstly, the U.S. matched multiple birth dataset 1995–2000 does not reflect current obstetric practices associated with higher cesarean delivery rates. However, the more liberal use of cesarean section in current obstetric practices may have allowed more low-risk women to undertake unnecessary cesarean sections, and thus may have somewhat compromised the benefits of cesarean delivery. Secondly, we did not have data on maternal death and severe birth-related morbidities such as severe hemorrhage, genital tract injury and hysterectomy. The optimal choice for mode of delivery needs to consider the risks and benefits for both the mothers and babies. The choice of optimal mode of delivery for twins needs to consider fetal presentation, especially the first-presenting twin. The first delivered twin might not be the first presenting twin in cesarean deliveries. Nevertheless, we observed similar findings when the analyses were restricted to twin sets with vertex presentation in both twins. Labor problems were used to capture which cesarean births were originally intended or planned as vaginal deliveries. We could have misclassifications of intended mode of delivery if the women had a trial of labor for vaginal delivery without labor problems, but took a cesarean section for other clinical indications–although the latter clinical risk factors might have been partly captured by the propensity score. Also, we have no data on chorionicity which could affect perinatal outcomes. However, this might have affected the comparisons less in favor of cesarean delivery since monochorionic twins are at higher risk for adverse outcomes, and would be more likely to be delivered by cesarean section than dichorionic twins. Furthermore, the lower risk of adverse perinatal outcomes in cesarean vs. vaginal deliveries at 36+ weeks gestation remained when the analyses were restricted to different-sex (dichorionic) twins. The study was retrospective and nonrandomized. We could not affirm that the observed associations are causal. We had no information on the clinical management protocols for twin pregnancies which might differ across hospitals; the analysis was based on routinely collected perinatal health data only. Lastly, we used propensity score to control for potential selection bias and confounding factors, but the adjustments might be partial due to the presence of unmeasured risk factors for cesarean delivery. Nevertheless, we noted that the adjustment for propensity score tends to produce results (HRs for the primary outcome) more in favor of cesarean delivery. Thus, more complete adjustment for other risk factors may tend to yield results even more in favor of cesarean delivery in twin pregnancies at ≥36 weeks gestation.

## Conclusion

Cesarean delivery may be beneficial for perinatal outcomes overall in twin pregnancies at or beyond 36 weeks gestation.
